# Mitochondrial transfer in solid tumors: dual roles in tumor progression and therapeutic targeting

**DOI:** 10.20517/evcna.2026.68

**Published:** 2026-08-12

**Authors:** Lirui Huang, Zeru Li, Cheng Qin, Bangbo Zhao, Tianyu Li, Yutong Zhao, Haoyu Shi, Xiangyu Zhang, Yiping Xie, Yutong Yan, Weibin Wang

**Affiliations:** Department of General Surgery, State Key Laboratory of Complex Severe and Rare Diseases, Peking Union Medical College Hospital, Chinese Academy of Medical Sciences and Peking Union Medical College, Beijing 100730, China.; ^#^These authors contributed equally to this work.

**Keywords:** Solid tumor, mitochondrial transfer, cell-to-cell communication, extracellular vesicles, tunneling nanotubes

## Abstract

Solid tumors account for the majority of cancer cases, and their incidence continues to increase. A solid tumor is not an isolated mass but interacts dynamically with cells in the tumor microenvironment. Mitochondria, which are essential for energy production and the regulation of apoptosis, play a critical role in cellular function and may serve as therapeutic targets. A growing body of evidence demonstrates that mitochondria can be transferred between cells via extracellular vesicles, tunneling nanotubes, gap junctions, and cell fusion. This process across various cell types, including stromal cells, immune cells, and cancer cells, and is enhanced under stress conditions, such as hypoxia and chemotherapy. Under these conditions, mitochondrial transfer enables tumor cells to acquire functional mitochondria from neighboring cells, thereby enhancing their metabolic plasticity and survival capacity. Mitochondrial transfer thus represents a critical adaptive mechanism that supports tumor progression. Targeting this dynamic form of intercellular communication offers a promising therapeutic strategy to overcome tumor metabolic plasticity, immunosuppression, and resistance to therapy.

## INTRODUCTION

The global burden of cancer continues to increase. According to the latest data from the Global Burden of Disease Study 2023 (GBD 2023), there were 18.5 million new cancer cases worldwide in 2023, of which 10.4 million resulted in death^[1]^. Solid tumors are among the primary contributors responsible for cancer-related mortality, and are distinguished by aggressive tumor proliferation, as well as local invasion and distant metastases. Remarkable therapeutic gains have been realized for both *in situ* disease management and non-metastatic survival thanks to modern developments in multimodal regimens, with surgical removal of the primary neoplasm serving as a core contributor^[2]^. An intricate interconnected system known as the tumor microenvironment (TME) consists of neoplastic cells and an array of supporting components, among which are endothelial cells, immune cells, stromal cells, and extracellular matrix^[3]^. From the initial phases of tumor development, reciprocal interactions between malignant cells and their surrounding niche contribute to tumor survival, local invasive growth, and metastatic propagation^[3]^.

Recently, accumulating evidence has shown that mitochondria not only play a critical role in host cells, but can also be transferred into recipient cells, where they regulate various cellular functions^[4]^. Mitochondrial transfer occurs across different tissues under both physiological and pathological conditions^[4]^. Released mitochondria may originate from both dying cells and viable cells^[5]^. Stress conditions, such as oxygen-glucose depletion, drug-induced oxidative stress and inflammation, can promote the release of damaged or fragmented extracellular mitochondria, thereby facilitating intercellular mitochondrial transfer^[6]^. More recently, accumulating evidence has shown that mitochondrial transfer can occur between different cell types. To date, four primary mechanisms underlying mitochondrial transfer have been identified: extracellular vesicles (EVs)^[7]^, tunneling nanotubes (TNTs)^[8]^, gap junctions (GJs)^[9]^ and cell fusion^[10]^. Physiological mitochondrial rescue is a transient homeostatic protective mechanism in normal tissues triggered by mild stress such as temporary hypoxia. Mitochondria move bidirectionally between adjacent normal cells to supplement impaired energy supply, and this transfer will stop automatically after cellular metabolic balance recovers; it only serves tissue repair without malignant risks. It has been suggested that the transfer of intact mitochondria may serve as a preventive mechanism in a variety of conditions requiring metabolic rescue, including acute myocardial infarction and stroke-induced ischemic injury^[11]^. The acquisition of functional mitochondria or their components may help restore or enhance mitochondrial function^[4]^. In contrast, tumor-promoting mitochondrial transfer is sustained pathological communication activated by long-term harsh tumor microenvironmental stress, including nutrient deprivation, persistent hypoxia, and chemotherapy damage. This transfer is predominantly unidirectional toward metabolically defective tumor cells. Recipient tumor cells acquire intact functional mitochondria to achieve metabolic remodeling, stronger invasive capacity and cross-drug resistance, ultimately accelerating tumor growth, metastasis and treatment failure^[12]^.

The transferred mitochondria may also impact other cellular structures. In the context of previous findingson mitochondrial transfer, functional mitochondria acquired by recipient tumor cells may integrate into thehost mitochondrial-associated endoplasmic reticulum (ER) membranes (MAMs) network, restore proper ER- mitochondrial Ca^2+^ homeostasis, and mitigate chronic ER stress, promoting chemoresistance and survival. Conversely, if transferred mitochondria fail to establish appropriate contact sites, they might exacerbate Ca^2+^ dysregulation and sensitize cells to apoptosis^[13]^. In solid tumors, the balance between mitochondrial biogenesis and mitophagy is often dysregulated, contributing to metabolic flexibility and therapy resistance^[14]^. Additionally, mitochondrial-derived vesicles (MDVs) can deliver oxidized mitochondrial components directly to lysosomes for degradation, representing an alternative quality control pathway^[14]^. In tumors with high basal autophagy (a common feature of many solid tumors), transferred mitochondria could be rapidly targeted for mitophagy and degraded, limiting their functional impact.

This review aims to provide a comprehensive synthesis of the complex biological functions of mitochondrial transfer in solid tumors, with a particular focus on its bidirectional and context-dependent mechanisms. Evidence for both pro-tumorigenic and potential anti-tumor effects is critically examined. Building on this foundation, the review systematically evaluates emerging intervention strategies-such as inhibiting detrimental transfer or enhancing beneficial transfer-and discusses the prospects and challenges for their clinical translation.

## MECHANISMS OF MITOCHONDRIAL TRANSFER IN CANCER CELLS

Up to now, four primary mechanisms underlying mitochondrial transfer have been identified: EVs, TNTs, GJs and cell fusion [Figure 1].

**Figure 1:**
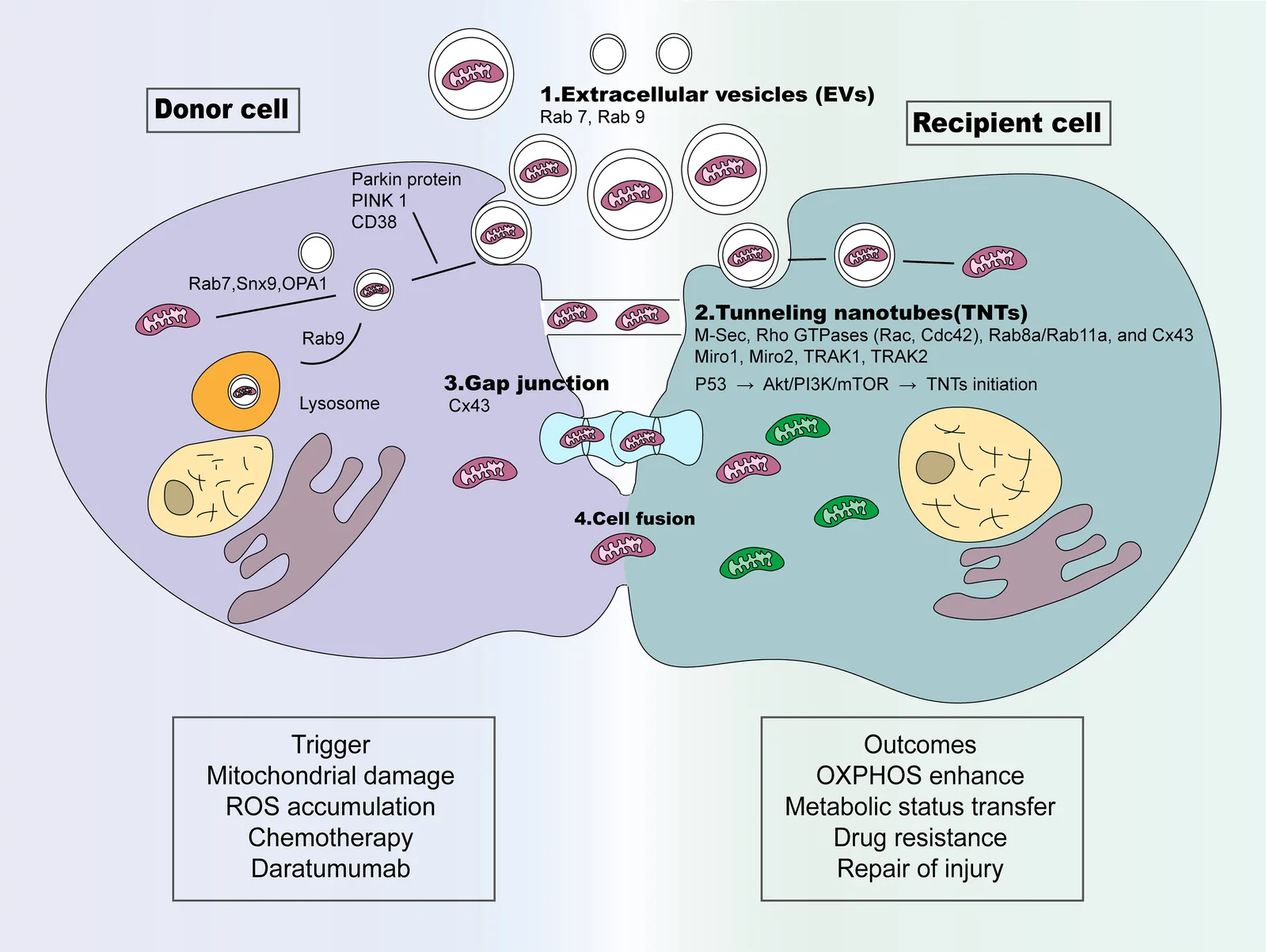
The ways of mitochondrial transfer in cancer cells. Four primary mechanisms underlying mitochondrial transfer have been identified: (1) EVs: Mitochondria-containing EVs form under the regulation of Snx9, Rab9 and OPA1, while Rab7 mediates EVs-lysosome fusion for cargo degradation. Alternatively, PINK1/Parkin signaling drives extracellular release of mitochondrial cargo-laden vesicles; (2) TNTs: Multiple signaling molecules coordinate TNT formation, including M-Sec, Rho GTPases (Rac, Cdc42), Rab8a/Rab11a, and Cx43 gap junction proteins. Miro1/2 together with TRAK1/2 form adaptor complexes that mediate mitochondrial transfer within microtubule-containing TNTs. TNT development is further modulated by stress and pro-survival cascades such as p53, PI3K/Akt, MAPK/ERK, and mTOR pathways; (3) GJ: Gap junctions are assembled by two hemichannels, each composed of six oligomerized connexin subunits, enabling direct molecular exchange between adjacent cells. Cx43 is the best-characterized connexin subtype supporting mitochondrial transfer; (4) Cell fusion: Increased cell fusion events between different cells enhance mitochondrial transfer. CD38: Cluster of differentiation 38; Cx43: connexin 43; M-Sec: Mammalian Sec14-like protein 2; Miro1: mitochondrial Rho GTPase 1; Miro2: mitochondrial Rho GTPase 2; OPA1: Optic atrophy 1; PINK1: phosphatase and tensin homolog-induced kinase 1; Rab11a: Ras-related protein 11a; Rab7: Ras-related protein 7; Rab8a: Ras-related protein 8a; Rab9: Ras-related protein 9; Rac: Rac family small GTPase; Snx9: Sorting nexin 9; TRAK1: trafficking kinesin protein 1; TRAK2: trafficking kinesin protein 2; EVs: extracellular vesicles; TNTs: tunneling nanotubes; GJ: gap junction; Cdc42: cell division control protein 42 homolog; p53: tumor protein p53; PI3K: phosphoinositide 3-kinase; Akt: protein kinase B; MAPK: mitogen-activated protein kinase; ERK: extracellular signal-regulated kinase; mTOR: mechanistic target of rapamycin.

### EVs

EVs are membranous structures enclosed by lipid bilayers that are produced and released by cells^[15,16]^, with sizes ranging from 30 to 1,000 nm^[15]^. Two dominant classes of EVs have been identified, namely microvesicles (MVs) and exosomes. The biogenesis of MVs depends on outward membrane budding, while exosome production originates from the cellular endocytic pathway^[17]^. A wide range of biomolecules are packaged within EVs, including proteins, lipids, and metabolites. These vesicles also harbor diverse genetic materials such as mRNA, non-coding RNA, nuclear DNA, and mitochondrial DNA, with sporadic innate organelle components observed in specific EV subsets. The transfer of intracellular cargo to recipient cells reshapes cellular biological phenotypes through the regulation of gene and protein expression profiles, as well as nutrient metabolic status, thereby disrupting cell homeostasis^[18]^. Cargo composition, coupled with the dynamic release and internalization patterns of EVs, collectively dictates their functional performance within the TME^[18]^. Accumulating experimental evidence obtained via transmission electron microscopy, immunofluorescence, and western blotting confirms the presence of mitochondrial components in EVs^[19]^. Mitochondria-derived vesicles (MDVs) typically range from 80 to 150 nm in diameter and are characterized by a double membrane structure lacking cristae^[19]^. The formation of MDVs requires sorting nexin 9 (Snx9) and Ras-related protein 9 (Rab9). Through the coordinated action of Snx9 and optic atrophy 1 (OPA1), mitochondria and their components are selectively incorporated into MDVs for degradation^[20]^. Ras-related protein 7 (Rab7) mediates the trafficking of MDVs to lysosomes^[21]^. In addition to being targeted to lysosomes, MDVs are capable of extracellular release, and this secretory process is regulated by two key molecules: the E3 ubiquitin ligase Parkin and PTEN-induced kinase 1 (PINK1)^[22]^. Notably, depletion of arrestin domain-containing protein 1 (ARRDC1) in mouse embryonic fibroblast cells markedly increases the abundance of mitochondrial proteins encapsulated in EVs, supporting the regulatory role of ARRDC1 in mediating mitochondrial protein incorporation into vesicle cargos^[23]^. CD38 catalyzes the production of the calcium-mobilizing messenger cyclic ADP-ribose (cADPR) within mitochondrial membranes^[24]^. Stimulation of astrocytes with cADPR activates CD38 signaling, resulting in increased levels of extracellular mitochondrial in conditioned media, along with enhanced mitochondrial function in a calcium-dependent manner^[11]^. Activation of the CD38/inositol 1,4,5-trisphosphate receptor (IP3R)/Ca^2+^ pathway generates “super donor” mesenchymal stem cells (MSCs) which produce EVs containing mitochondrial (EV-Mito) at threefold higher levels than those normal MSCs^[25]^. Inhibition of CD38 reduces extracellular mitochondrial levels and their astrocyte-to-neuron transfer^[11]^. Furthermore, pharmacological inhibition of MV formation using GW4869 resulted in a decrease in the extracellular release of mitochondria^[26]^.

### TNTs

TNTs serve as intercellular channels enabling communication between spatially separated cells in the same tissue, even spanning distances up to hundreds of micrometers^[27]^. Two primary mechanisms for the formation of TNTs have been proposed^[27,28]^. The first mechanism is largely influenced by cell movement and takes place when cells initially in close proximity subsequently separate^[27]^. The second mechanism involves the extension and fusion of actin filament-containing membrane protrusions. These protrusions originate from the donor cell and fuse with the target cell’s membrane, without requiring cell motility or prior contact^[27]^. As a direct form of cell-to-cell communication, TNTs have been reported in several pathological conditions, including cancer, in both *in vitro* and *in vivo* models^[29]^. Two types of TNTs based on the cytoskeletal components involved in their formation^[30]^. One subset comprises thin filaments composed solely of F-actin, whose formation can be induced by the mammalian protein M-sec. A thicker subset, measuring 0.7 μm, known as microtubule-bearing TNTs, contains both F-actin and microtubules^[30]^. It may facilitate kinesin- and dynein-dependent transport of mitochondria between interconnected cells^[8]^. The movement of mitochondria along microtubules is enabled by a protein complex containing the calcium-sensitive adaptor protein mitochondrial Rho GTPase 1 (Miro1, encoded by RHOT1). Miro1, along with associated factors such as Miro2, trafficking kinesin-binding protein 1 (TRAK1), TRAK2, and myosin XIX (Myo19), is essential for regulating mitochondrial transport^[31]^. Miro family proteins modulate mitochondrial transfer along TNTs, acting as molecular adaptors to connect mitochondria with cytoskeletal regulatory proteins^[32]^. They are also crucial for actin rearrangement via Guanosine diphosphate (GDP)-guanosine triphosphate (GTP) cycling.^[27]^. The downstream effectors of Miro are essential to this process because they regulate actin polymerization via Arp2/3 nucleation and include the Wiskott-Aldrich syndrome protein (WASP) and WASP family verprolin-homologous 2 (WAVE2). Inhibition of the Arp2/3 complex resulted in decreased formation of tumor microtubes in pancreatic cancer cells, highlighting the significant role of actin polymerization in their development^[33]^. Rac and Cdc42, two pivotal members of the Rho GTPase family responsible for actin cytoskeleton polymerization, are essential for TNT generation. Localization of these proteins within TNTs of T cells further confirms their regulatory role, and targeted Cdc42 inhibition is sufficient to hinder TNT formation^[34]^. Another study demonstrated that Rab8a/Rab11a regulate intercellular communications between neural cells via TNTs^[35]^. Knockdown of Rab8a/Rab11a promotes apoptosis in Schwann cells and inhibits their migration by suppressing TNT formation^[35]^. The integral GJ protein, connexin 43 (Cx43), which is present in TNT-like structures, plays a key role in communication between TNT-connected cells^[36]^. Knockdown of Cx43 has been reported to significantly affect TNT formation and reduce mitochondrial transfer between MSCs and epithelial cells^[37]^. The biogenesis of TNTs involves various stress-signaling pathways, such as p53 and mitogen-activated protein kinase (MAPK), alongside pro-survival mechanisms, including epidermal growth factor receptor (EGFR), protein kinase B (Akt), Rho-associated coiled-coil kinase (ROCK), p21-activated kinase (PAK), MAPK/extracellular signal-regulated kinase (ERK), phosphoinositide 3-kinase (PI3K), or mechanistic target of rapamycin (mTOR)^[6]^. Signaling pathway activation ultimately targets effector proteins that mediate membrane recycling processes and cytoskeletal dynamic remodeling^[6]^. tumor microtubes (TMs) constitute a type of tubular structure different from TNTs. TNTs are thin, transient intercellular channels composed exclusively of actin filaments and are ubiquitously present in all mammalian cell types. By contrast, TMs are thick, extraordinarily stable membrane protrusions characterized by the coexistence of F-actin and uninterrupted microtubule bundles^[38]^.

### GJs

GJs constitute direct intercellular routes that enable molecular exchange between neighboring cells. They are established through the connection of hemichannels from each cell, which are hollow tube-like structures resulting from the oligomerization of six connexin subunits in the plasma membrane^[39]^. GJs also represent an important mechanism for mitochondrial transfer, although fewer studies have examined GJs compared with TNTs. This is partly because TNTs are more readily observed in *in vitro* co-culture systems^[40]^. Connexin family proteins play crucial roles in the formation of GJs. Among these, Cx43 is the most extensively studied. Latest studies reveal that the reactive oxygen species (ROS)-mediated oxidative stress response during emergency granulopoiesis initiates PI3K-Akt activation, thereby opening Cx43 channels and supporting intercellular mitochondrial transfer from bone marrow mesenchymal stem cells (BMSCs) to hematopoietic stem cells^[41]^. Mitochondrial transfer occurs exclusively when GJs operate normally at areas of close contact between cells^[42]^. One study indicates that during the process of GJ internalization, one cell engulfs small portions of a neighboring cell, facilitating mitochondrial transfer^[43]^. Emerging evidence indicates that GJs strengthen the adhesive interaction between TNTs and recipient cells, while simultaneously enhancing EV internalization efficiency^[44]^. Following treatment with the GJ enhancer retinoic acid, mitochondrial transfer was increased, which means that GJs participated in the transfer of mitochondria from BMSCs to VSC 4.1 motor neurons^[9]^. In glioblastoma, GJs formed by Cx43 may facilitate TNT formation and enhance mitochondrial transfer^[45]^. This may occur by enabling tumor microtubules to establish contact with adjacent cells, thereby promoting increased signal exchanges and ultimately advancing tumor progression^[45]^.

### Cell fusion

Cell fusion involves the fusion of the plasma membranes between two separate cells. Such intercellular fusion facilitates the exchange of cytosolic substances and organelles, whereas the nuclei of the two cells remain structurally distinct^[46]^. Owing to the low prevalence of cell fusion events in normal higher eukaryotic physiology, this process is not classified as a primary route for mitochondrial intercellular transfer^[40]^. Some studies suggest that the events described above may represent partial or transient cell fusion, whereas permanent cell fusion results in hybrid cells that share cytoplasm and often exhibit abnormal karyotypes. This leads to a more complex interplay of mitochondrial dynamics, including transfer, fusion, and fission events^[10]^. Increased cell fusion events between MSCs and differentiated cells enhance mitochondrial transfer and promote tissue regeneration, as evidenced by the detection of heterokaryons in regenerated tissues^[47]^. Primary glioblastoma cells can acquire mitochondria through phagocytosis of tumor-activated stromal cells, utilizing cytosolic components of these cells for their own benefit^[48]^. Similarly, mitochondrial transfer mediated by cell fusion has been observed in multiple systems. For instance, mitochondria from human stem cells transferred to mature cardiomyocytes persist over time in mouse cardiac progenitor-like cells generated via cell fusion^[49]^. In another example, mitochondrial transfer between BMSCs and myeloma cells is bidirectional and occurs through partial cell fusion, and this process can be enhanced significantly with chemotherapeutic drugs. This process leads to enhanced survival and elevated adenosine triphosphate (ATP) levels, as well as reduced mitochondrial superoxide levels^[50]^. BMSC-mediated mitochondrial transfer to Müller cells occurs via cell fusion. Such intercellular mitochondrial supplementation restores mitochondrial activity, reduces oxidative damage, and suppresses gliotic responses^[51]^.

## PATTERNS OF MITOCHONDRIAL TRANSFER IN CANCER

Mitochondrial transfer among tumor cells predominantly enhances their resistance to stress conditions. Recent research has highlighted the dynamic exchange of mitochondria within the TME, occurring among different cell types [Table 1 and Figure 2]^[4]^.

**Figure 2:**
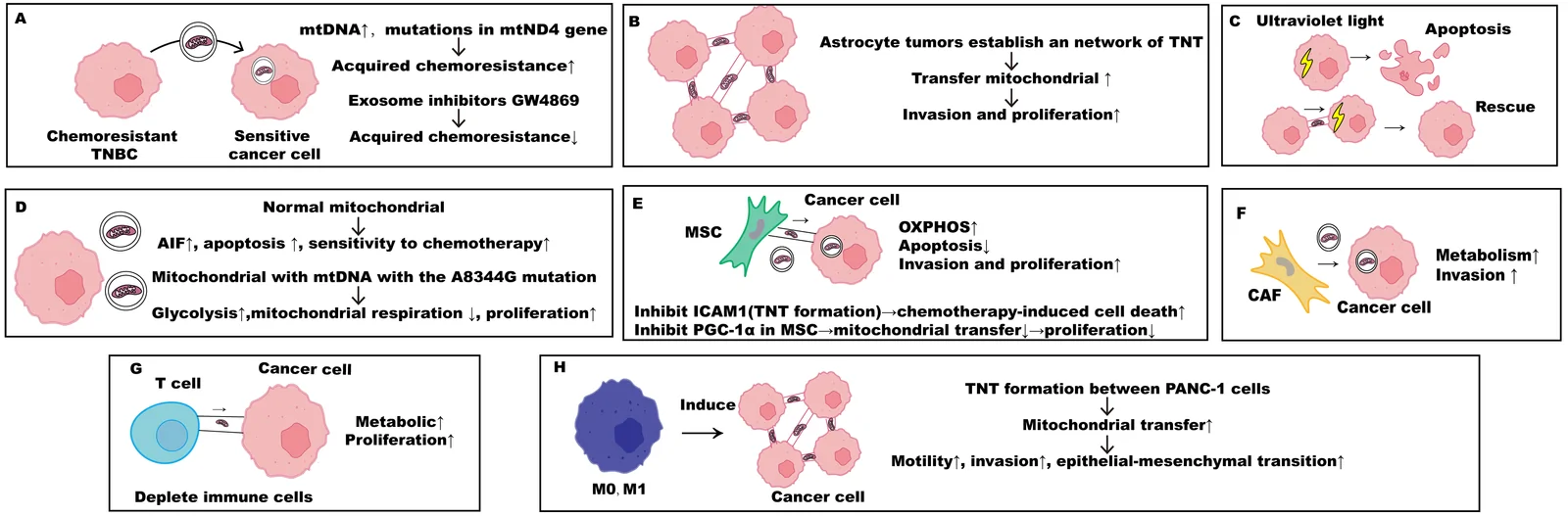
Patterns of mitochondrial transfer in cancer. (A-C) Mitochondrial transfer among cancer cells. (A) Chemoresistant TNBC cells transfer mitochondria to sensitive cancer cells by EVs; (B) Astrocyte tumors establish an interconnected network of TNTs and tumor microtubules and can transport mitochondria, which leads to increased tumor invasion and proliferation; (C) UV-induced stressed rat pheochromocytoma cells formed TNTs with normal cells and obtained transferred mitochondria that participate in the rescue effect; (D-F) Mitochondrial transfer between cancer and stromal cells. (D) MCF-7 breast cancer cells can internalize complete normal mitochondria and manifest as inhibition of cell proliferation; (E) Mitochondria extracted from MSCs to cancer cells result in an enhancement of OXPHOS activity that promotes the proliferative and invasive characteristics of recipient cancer cells. Inhibition of ICAM-1 blocks TNT formation, which promotes chemotherapy-induced cell death; Inhibition of PGC-1α in MSC suppresses mitochondrial transfer and impairs tumor cell proliferation; (F) CAFs transfer mitochondria to prostate cancer cells, further enhancing their metabolic and motile features; (G and H) Mitochondrial transfer between cancer and immune cells. (G) Mitochondrial transfer from T cells to cancer cells, facilitated by nanotubes, enhances the metabolic capabilities of the cancer cells while simultaneously depleting the immune cells; (H) M0 and M1 macrophages induced TNT network formation in PANC-1 cells, mitochondria delivered through TNTs. AIF: Apoptosis-inducing factor; CAF: cancer-associated fibroblast; ICAM-1: intercellular adhesion molecule-1; M0: M0-macrophage; M1: M1-macrophage; MSC: mesenchymal stem cell; mtDNA: mitochondrial deoxyribonucleic acid; OXPHOS: oxidative phosphorylation; PGC-1α: peroxisome-proliferator-activated receptor-gamma coactivator-1α; TNBC: triple-negative breast cancer; TNT: tunneling nanotube; EVs: extracellular vesicles; TNTs: tunneling nanotubes; UV: ultraviolet light.

**Table 1:** The mechanism and effects of mitochondrial transfer

**Donor cells**	**Recipient cells**	**Transfer approach**	**Mechanism (signaling pathways, molecules, and structural basis)**	**Effect on recipient cells**	**Ref.**
CAFs	Prostate cancer cells	TNT	Lactate shuttle from CAFs → intracellular NAD+/NADH ratio ↓ → activation of SIRT1 deacetylase → PGC-1α activation → mitochondrial biogenesis; Concurrent direct transfer of whole mitochondria via TNTs	Mitochondrial mass and oxidative activity ↑; TCA cycle dysregulation; oncometabolite accumulation; altered ETC complex expression; superoxide generation ↑; proliferation, invasion & malignancy ↑	[60]
Immune Cells (CD8 T cells, NK cells, others)	Melanoma cells, breast cancer cells, colon adenocarcinoma	TNT	Mitochondrial hijacking; Exogenous mitochondrial fuse with endogenous networks → mtDNA leakage into cytosol → cGAS/STING pathway activation → Type I interferon-mediated immune evasion	Immune function (antigen presentation, NK/CD8 activation) ↓; Lymph node metastasis ↑; cGAS/STING/IFN pathway activation promotes immune evasion	[70]
Melanoma cells	Melanoma cells	TNT	Oxidative stress (ROS accumulation) → M-sec-dependent TNT formation → Miro-1-dependent mitochondrial transfer → Cargo (IR780) hitchhikes on mitochondrial for cotransport along TNTs	TNT network formation ↑; Deep tumor penetration of cargo bypassing physical barriers; Enhanced photodynamic killing upon NIR irradiation; Controllable drug delivery via TNT regulation; High biocompatibility for precision cancer therapy	[83,84]
Cancer cells (including melanoma, breast cancer, skin squamous cell carcinoma)	Tumor-infiltrating lymphocytes (TILs, primarily CD8+ T cells)	TNT, EV	Mitochondrial transfer via TNTs and small EVs; Co-transfer of mitophagy-inhibitory molecules USP30 (deubiquitinating enzyme inhibiting Parkin-mediated mitophagy) attached to cancer cell mitochondria → Transferred mitochondria resist ROS-induced mitophagy → Homoplastic replacement of endogenous T cell mtDNA; USP30 inhibitor CMPD-39 or siUSP30 partially prevents replacement	Impaired antitumor immunity: Basal respiration and OXPHOS, ATP ↓; ROS production ↑; Membrane potential ↓; Senescence (β-galactosidase, p16, p53) ↑; Central memory formation (CCR7^+^CD45RA^low^, KLRG1^low^) ↓; Effector activation (PD-1, CD69) ↓; Apoptosis ↑	[71]
Cancer-associated neurons	Breast cancer cells	TNT	Metabolic reprogramming of neurons when co-cultured with cancer cells ↑ → neuronal mitochondrial mass → Direct transfer of functional mitochondria via TNTs	Metabolic capacity, stemness, and resistance to metastatic stressors (oxidative/shear stress) ↑; Selective enrichment of mitochondrial-receiving cancer cells at metastatic sites	[65]
Astrocytes	Glioblastoma cells	TNT	GAP43-dependent intercellular connections (first identification of GAP43 in mitochondrial transfer; GAP43 knockdown significantly reduces transfer); TM-mediated transfer (network-forming intercellular connections)	Basal and maximal respiration ↑; mitochondrial biology genes (ETC components) ↑; ATP5A expression ↑; ATP levels; cell cycle progression to G2/M ↑; self-renewal ↑; tumorigenicity ↑	[64]
BMSC	CD8+ T cells	TNT	TNT-mediated connections (nanotube structures visualized by field-emission SEM; mitochondria confirmed inside nanotubes by confocal); Talin 2-dependent on both donor and recipient cells (optimal transfer requires Talin 2)	Mitochondrial content ↑; mitochondrial respiration ↑; spare respiratory capacity ↑; tumor infiltration ↑; expansion *in vivo* ↑; exhaustion markers ↓; antitumor efficacy ↑; enhanced CAR-T function	[8]
NKT cells	Breast cancer cells	TNT	TNT formation (actin-based nanotubes visualized by SEM); Protein farnesylation/geranylgeranylation-dependent assembly (inhibited by L-778123 targeting FTase/GGTase1); Miro-1-dependent mitochondrial transferring along cytoskeleton; Exocyst complex (Sec3/Sec5) localization to TNT assembly sites	Cancer cell oxidative metabolism ↑; Antitumor immune cell viability and function ↓; Immune evasion; TNT inhibition + anti-PD-1 improves outcomes	[67]
Macrophage	Breast cancer cells	TNT	Macrophage-derived inflammatory mediators → enhanced TNT formation; TNT-mediated release of “microplasts” (independently migrating viable cytoplasmic fragments); Actin cytoskeleton-dependent (inhibited by cytochalasin-B); Dynamic TNT retraction and microplast resorption	Formation of TNT-based intercellular networks; mitochondrial transfer to microplasts; expanded intercellular communication range; organelle redistribution across cell population	[69]
Glioma cells	Glioma cells	TNT, TM	TM formation; Ultra-long membrane protrusions; Gap junction-mediated communication; 1p/19q status-dependent	Formation of interconnected malignant network; Resistance to cytotoxic therapies	[54]
Chemo-resistant breast cancer cellsMDA-MB-231	Chemo-sensitive breast cancer cells	EV	Primarily EV/exosome-mediated (blocked by GW4869); Transfer of mutant mtND4 mtDNA; TNTs as secondary route	Acquired chemoresistance; mutant mtDNA copy number ↑; Propagation of resistant phenotype	[56]
Glioblastoma stem-like cells	Glioblastoma stem-like cells	TNT	TNT formation in 2D and 3D organoids; TM in 3D organoids; Heterogeneous stress responses to radiation; Open-ended functional TNTs	Formation of interconnected networks; heterogeneous radiation responses; potential contribution to therapy resistance	[45]
Macrophages	Breast cancer cells	NA	Cell contact-dependent transfer; Fragmented mitochondrial network in pro-tumorigenic macrophages → higher transfer rates; Donor mitochondrial dysfunction promotes transfer	Transfer of dysfunctional mitochondria → ROS accumulation → ERK activation; proliferation independent of bioenergetics ↑	[68]
MSCs	Gastric cancer cells	EV	OXA administration → GC cells actively secrete ECM → matrix stiffness in tumor microenvironment ↑; Mechanical signals (ECM stiffness) promote MSCs to transfer mitochondria to GC cells via MVs; RhoA/ROCK1 pathway involved (inhibition alleviates OXA resistance)	Repair of mitochondrial function; mitophagy ↓; chemotherapy resistance	[7]
Isolated from HeLa-DsRed2-mito cells	Hepatocellular carcinoma cells, fibroblasts	Endocytosis	Endocytosis-mediated mitochondrial engulfment (blocked by macropinocytosis inhibitors EIPA and cytochalasin D); Requires intact mitochondrial outer membrane proteins; Heparan sulfate proteoglycan-dependent recognition (blocked by heparin/pentosan polysulfate)	Co-localization with endogenous mitochondrial network; Functional integration into existing network	[86]
Astrocytes	Glioma cells	Endocytosis	Serum-glucose starvation → NAD+ accumulation and release → extracellular NAD+ catalyzed by CD38 (NAD+ glycohydrolase) → generation of intracellular cADPR → Ca^2+^ release from ER stores → cytoskeleton remodeling and plasma membrane invagination → endocytosis-mediated internalization; TNT formation; Miro-1-dependent mitochondrial transferring along cytoskeleton; Exocyst complex (Sec3/Sec5) localization to TNT assembly sites	TCA cycle gene/protein expression ↑; aerobic respiration ↑; glycolysis (Warburg effect reversal) ↓; cytochrome c release ↑; cleaved caspase 9 ↑; proliferation ↓; radiosensitivity ↑; tumor growth *in vivo* ↓	[62]
Pancreatic cancer cells	Pancreatic cancer cells	TNT	Macrophage-derived soluble factors induce TNT formation in cancer cells; TNT-mediated mitochondrial transfer facilitation	Motility ↑; Invasion ↑; EMT ↑; Facilitation of mitochondrial transfer	[52]

↑ Indicates promotion, elevation or increase, while ↓ indicates inhibition, reduction or decrease. ATP: Adenosine triphosphate; BMSC: bone marrow mesenchymal stem cell; CCR7: C-C motif chemokine receptor 7; cADPR: cyclic adenosine diphosphate ribose; cGAS: cyclic GMP-AMP synthase; CMPD-39: USP30 inhibitor compound 39; ECM: extracellular matrix; EMT: epithelial-mesenchymal transition; EIPA: 5-(N-ethyl-N-isopropyl)amiloride; ER: endoplasmic reticulum; ERK: extracellular regulated protein kinase; ETC: electron transport chain; EV: extracellular vesicle; FTase: farnesyltransferase; GAP43: growth-associated protein 43; GC: gastric cancer; GGTase1: geranylgeranyl transferase I; HeLa-DsRed2-mito: HeLa cell line stably expressing DsRed2-labelled mitochondria; Miro-1: mitochondrial Rho GTPase 1; mtDNA: mitochondrial deoxyribonucleic acid; mtND4: mitochondrial NADH dehydrogenase subunit 4; MV: microvesicle; NAD+: oxidized nicotinamide adenine dinucleotide; NADH: reduced nicotinamide adenine dinucleotide; NK cell: natural killer cell; NKT cell: natural killer T cell; OXA: oxaliplatin; OXPHOS: oxidative phosphorylation; p16: cyclin-dependent kinase inhibitor p16; p53: tumor protein p53; PD-1: programmed cell death protein 1; PGC-1α: peroxisome proliferator-activated receptor gamma coactivator 1-alpha; RhoA: Ras homolog family member A; ROCK1: Rho-associated coiled-coil containing protein kinase 1; ROS: reactive oxygen species; Sec3/Sec5: exocyst complex subunits Sec3 and Sec5; SEM: scanning electron microscopy; SIRT1: sirtuin 1; STING: stimulator of interferon genes; TCA: tricarboxylic acid; TIL: tumor-infiltrating lymphocyte; TM: tumor microtubule; TNT: tunneling nanotube; USP30: ubiquitin-specific protease 30; α-SMA: alpha-smooth muscle actin; CAFs: cancer-associated fibroblasts; MVs: microvesicles.

### Mitochondrial transfer among cancer cells

The main effect of mitochondrial transfer among tumor cells is to enhance their resistance to stress conditions. A study showed that conditioned media from M0 and M1 macrophages derived from human acute monocytic leukemia (THP-1) cells promote TNT formation in human pancreatic ductal adenocarcinoma (PANC-1) cancer cells. Mitochondria and lysosomes were observed to be transported through these TNTs. TNT formation was closely associated with increased cell motility and the induction of epithelial-mesenchymal transition (EMT) in PANC-1 pancreatic cancer cells^[52]^. Similarly, ultraviolet light-stressed PC12 rat pheochromocytoma cells form TNTs with unstressed PC12 cells and acquire mitochondria that contribute to a rescue effect^[53]^. TNT-mediated transfer of functional mitochondria can reverse cellular stress during early stages of apoptosis^[53]^. These findings suggest that cancer cells may enhance their proliferative capacity by exchanging mitochondria to overcome stress conditions. Chemo-resistant triple-negative breast cancer (TNBC) cells can transfer mitochondria to sensitive TNBC cells via EVs, thereby promoting chemoresistance^[54]^. This process is associated with elevated mtDNA levels, including mutations in the *mtND4* gene that contribute to tumorigenesis^[54]^. Inhibition of mitochondrial transfer using exosome inhibitors such as GW4869 reduces the acquisition of chemoresistance in TNBC^[54]^. Astrocytic tumors establish interconnected networks of TNTs and tumor microtubules that facilitate mitochondrial transport^[55]^. Tumor microtubules act as key structural elements supporting glioma cell invasion and proliferation, thereby promoting efficient brain colonization^[55]^. Human breast adenocarcinoma (MCF-7) cells can internalize intact mitochondria from 143B osteosarcoma cybrids through simple co-culture. Such alterations restrain proliferative activity, initiate apoptosis-inducing factor (AIF)-mediated caspase-independent apoptosis, and markedly improve the responsiveness of tumor cells to chemotherapy treatment^[56]^. Furthermore, replacement with dysfunctional mitochondria harboring the A8344G mtDNA mutation (Mito8344) shifts cellular metabolism toward a glycolytic phenotype, driven by enhanced glycolysis and impaired mitochondrial respiration^[56]^.

### Mitochondrial transfer between cancer and stromal cells

Like other stromal cells, MSCs exhibit altruistic behavior in tumor formation, including the transfer of mitochondria to tumor cells. Tumor cells can establish TNT connections with endothelial cells and MSCs, which supports the bidirectional exchange of cytoplasmic cargoes encompassing miRNAs and mitochondria^[57]^. The transfer of limited quantities of MSC-derived mitochondria to cancer cells enhances oxidative phosphorylation, thereby promoting the proliferative and invasive capacities of recipient cells during co-incubation with exogenous mitochondria^[58]^. However, mitochondrial transfer from MSCs to osteosarcoma cells appears to occur primarily when the recipient cells are entirely depleted of mitochondria following pretreatment with ethidium bromide or rhodamine. Suppression of peroxisome proliferator-activated receptor gamma coactivator 1-alpha (PGC-1α) in BMSCs decreases the mitochondrial transfer, thereby limiting the proliferative potential of the tumor^[59]^. The transfer of mitochondria by MSCs to tumor cells also promotes drug resistance in tumor cells. For example, following oxaliplatin treatment, gastric cancer cells restructure the extracellular matrix, enhancing its stiffness, and stimulate the Ras homolog family member A (RhoA)/Rho-associated coiled-coil containing protein kinase 1 (ROCK1) pathway in MSCs, which facilitates mitochondrial transfer from MSCs to gastric cancer cells. Pharmacological treatment with the ROCK inhibitor Y-27632 decreases EVs‐dependent mitochondrial transfer and reinstates the tumor‐suppressive efficacy of oxaliplatin^[7]^.

Another type of stromal cell, cancer-associated fibroblasts (CAFs), also represents an important source of mitochondria for tumor cells. CAFs deliver mitochondria to prostate cancer cells, thereby enhancing their metabolic and migratory capacities^[60]^. In particular, CAFs enhance mitochondrial function in prostate cancer cells through activation of the lactate/sirtuin 1 (SIRT1)/PGC1-α axis and other mitochondrial-related signaling. This leads to the accumulation of tricarboxylic acid cycle intermediates, such as citrate, succinate, and fumarate, as well as the emergence of a mitochondrial reactive oxygen species (mtROS)-dependent signature, including the oxidation of Src and pyruvate kinase M2 (PKM2)^[60]^. Similarly, mitochondrial transfer from CAFs increases migration in aggressive breast cancer^[61]^.

Numerous studies have reported that astrocytes in the nervous system can promote glioma progression through mitochondrial transfer. In glioblastoma cells, it was found that tumor-activated stromal cells can transfer mitochondria to tumor cells through EVs^[48]^. Cancer cells lacking mtDNA (ρ0 cells) exhibit a significant delay in tumor formation compared to their parental counterparts; however, this growth impairment can be reversed through the acquisition of functional mitochondria from parental cells via mitochondrial transfer^[48]^. In addition, mitochondria derived from human astrocytes can be transferred to starved human glioma cells through co-incubation^[62,63]^. This process restores aerobic respiration and enhances sensitivity to radiotherapy^[62]^. Growth-associated protein 43 (GAP-43) has been implicated in facilitating mitochondrial transfer via tumor-astrocyte tumor microtubes^[64]^. Acquisition of astrocytic mitochondria facilitates the elevation of mitochondrial respiratory activity, while simultaneously boosting metabolic programs that promote tumor cell proliferation and malignant progression^[64]^. Tumor-associated neurons can transfer functional mitochondria to cancer cells in a contact-dependent manner through TNTs. Neuron-derived mitochondria integrate into the metabolic network of cancer cells to potentiate oxidative phosphorylation and ATP production, while reinforcing cellular redox homeostasis by increasing reduced glutathione levels. This mitochondrial reprogramming endows cancer cells with enhanced metabolic plasticity, stemness, and proliferative capability. Furthermore, the acquired mitochondria strengthen cancer cell resistance to oxidative stress and shear stress, facilitating the survival and colonization of circulating tumor cells in the bloodstream and distant tissue microenvironments. Lineage tracing evidence demonstrates that cancer cells acquiring neuronal mitochondria are significantly enriched in lung and brain metastatic foci, thereby conferring prominent metastatic advantages^[65]^.

### Mitochondrial transfer between cancer and immune cells

Mitochondrial uptake from immune cells enables tumor cells to proliferate aggressively and outcompete adjacent normal cells. Intercellular nanotubes serve as critical channels for mitochondrial shuttling from BMSCs to CD8+ T cells. This transfer process is governed by Talin2 (TLN2), a cytoskeletal protein responsible for actin assembly through the regulation of integrin-membrane protrusion interactions^[8]^. The transfer of mitochondria from BMSCs improves the metabolic capacity and antitumor immune response of CD8^+^ T cells against solid tumors. More significantly, the benefits resulting from mitochondrial transfer are not only immediate but also sustained over a longer duration^[8]^. Cancer cells acquire mitochondria from adjacent T cells, and this process can be tracked and quantified by single-cell RNA sequencing (RNA-seq) technology^[66]^. A classic study has shown that TNTs facilitate the transfer of mitochondria between breast cancer cells and T lymphocytes^[67]^. Mitochondrial tracing and quantitative metabolic analyses reveal that mitochondrial transfer from T cells to breast cancer cells boosts the metabolic fitness of cancer cells while concurrently weakening the immune cells^[67]^. Furthermore, the combined use of a farnesyltransferase inhibitor and a geranylgeranyl transferase I inhibitor effectively suppresses TNT formation and mitochondrial transfer. Meanwhile, treatment with a programmed cell death protein 1 (PD-1) immune checkpoint inhibitor has been shown to enhance antitumor effects in an aggressive immunocompetent breast cancer model^[67]^. Human breast cancer cells can acquire mitochondria from T cells and macrophages^[66,68]^. Macrophages have been shown to stimulate the formation of TNTs and microplasts (independently migrating viable cytoplasmic fragments) in cancer cells, leading to the development of extensive intercellular networks. Mitochondria can be packaged into microplasts and transported between cells via TNTs^[69]^. Cancer cells can seize mitochondria from multiple types of immune cells. Mitochondrial depletion in immune cells compromises their antigen presentation capability and costimulatory molecular machinery, accompanied by diminishing vitality of immune cells and cytotoxic potency of natural killer cells and CD8^+^ T cells. Upon internalization by cancer cells, exogenous mitochondria integrate with endogenous mitochondrial networks, leading to cytosolic mtDNA leakage. This process further activates the cyclic GMP-AMP synthase (cGAS)-stimulator of interferon genes (STING) signaling axis and induces type I interferonmediated immune evasion programs. Pharmacological blockade of mitochondrial transfer machineries, such as cGAS, STING, or type I interferons, effectively inhibits tumor lymph node metastasis. These observations demonstrate that tumor cells hijack immune cell mitochondria to abrogate antitumor immunosurveillance, while utilizing the acquired mitochondrial functions to meet the immunological demands of lymph node colonization and metastatic progression^[70]^. Tumor cells employ TNTs and EVs as critical transport channels to deliver mtDNA-mutated mitochondria to tumor-infiltrating lymphocytes in the tumor microenvironment. The persistent replacement of endogenous T-cell mitochondria by these aberrant exogenous organelles induces mitochondrial homogenization, a key mechanism underlying cancer immune evasion. Notably, these cancer-derived mitochondria carry the autophagy suppressor USP30, enabling them to resist intracellular ROS-triggered mitophagy and stably persist in T cells. The retained mutated mitochondria further induce T cell mitochondrial dysfunction (reduced membrane potential and defective oxidative phosphorylation) and excessive ROS accumulation, thereby disrupting T cell metabolism, accelerating cellular senescence, inhibiting proliferation, and impairing T cell cytotoxic effector function and memory formation, which ultimately blunts anti-tumor immune responses^[71]^.

In summary, research on mitochondrial transfer in tumor cells should consider the TME and the dynamic interactions between tumor cells and surrounding cell populations. This is crucial for understanding how mitochondrial transfer contributes to tumor progression and for developing strategies to prevent tumor cells from exploiting this process^[72]^.

## MITOCHONDRIAL TRANSFER AS A STRATEGY FOR SOLID TUMOR THERAPY

In recent years, the organelle-specific delivery of bioactive molecules has been utilized in cancer therapy to enhance specificity, improve treatment efficacy, minimize side effects, and reduce chemoresistance. Approaches targeting mitochondria enable direct manipulation of the mitochondrial membranes and components, as well as mitochondrial metabolism and mitochondria-associated apoptotic or regulatory signaling pathways^[73]^.

With increasing recognition of intercellular mitochondrial transfer, its potential applications are expanding across various medical disciplines [Table 2]. While spontaneous mitochondrial transfer within the TME typically promotes tumor invasion, the exogenous transplantation of healthy mitochondria into cells can exert opposing effects. Tumor cells employ TNTs and extracellular EVs as critical transport channels to deliver mtDNA-mutated mitochondria to tumor-infiltrating lymphocytes in the tumor microenvironment. The persistent replacement of endogenous T-cell mitochondria by these aberrant exogenous organelles induces mitochondrial homogenization, which serves as a key mechanism underlying cancer immune evasion. The therapeutic potential of targeting intercellular mitochondrial transfer lies in its promise as a novel anti-cancer strategy and its capacity to enhance cell-mediated immunotherapies. Future therapeutic approaches should aim to promote the cell-to-cell organelle transfer when it confers beneficial effects, or inhibit it when it supports tumor progression. Such strategies are particularly relevant for containing neoplastic cells and reducing their associated chemoresistance^[74]^.

**Table 2:** The strategies for targeted mitochondrial delivery and mechanisms

	**Mechanism**	**Ref.**
**Agents inhibiting mitochondrial transfer**		
Taxanes	Inhibit formation of TNT	[29]
Vinca alkaloids	Inhibit formation of TNT	[29]
Latrunculin	Actin-disrupting agents, inhibit formation of TNT	[75]
Cytochalasin B and D	Actin-disrupting agents, inhibit formation of TNT	[75]
L-778,123	Inhibitor of farnesyltransferase and geranylgeranyl transferase I, inhibits formation of TNT	[67]
Belamaf	Microtubule-disrupting agent	[77]
18-α-glycyrrhetinic acid	Inhibitors targeting gap junctions	[79]
Dynasore	Inhibits vesicular endocytosis	[79]
**Mitochondrial transplantation**		
MitoCeption	Mitochondria entering recipient cells through internalization induced by centrifugation and heat shock during co-culture	[58]
Transplantation of mitochondria into prostate cancer cells and ovarian cancer cells	Increased apoptosis in these cells as well as enhancing the sensitivity of mice to chemotherapy	[92]
Transplantation of mitochondria into melanoma-bearing mice	General chromosome silencing and mitochondrial activity increasing	[93]
Mitochondrial transplantation in hepatocellular carcinoma	Decreased glycolysis, promoted dephosphorylation of p-Bad, downregulated the expression of Bcl-2, increased Bax	[94]
Mitochondria isolated from healthy mouse livers transferred into melanoma model mice	Reduced glycolysis and produced an oxidative intracellular environment, inhibiting tumor growth and prolonging the survival duration of the animals	[95]
Transplanted normal human GES-1 mitochondria to human gastric AGS	Reduced the expression of mesenchymal markers α-SMA, MMP-9, snail, vimentin and N-cadherin, whereas the epithelial markers E-cadherin and claudin-1 were not changed. The proteins implicated in the cell cycle, such as cyclin B1 and D1, were decreased	[96]
**Promote spontaneous transfer of mitochondria**		
Promote the formation of TNT between tumor cells and facilitate mitochondrial transfer through siPINK1	PINK1-mediated mitophagy inhibition enhances mitochondrial intercellular transport by increasing mitochondrial motility, oxidative phosphorylation, and TNT formation. Engineered mitochondria carrying photosensitizer IR780 achieve deep tumor penetration via TNTs, and subsequent NIR irradiation triggers potent photodynamic killing while disrupting the transfer network, achieving 43% complete tumor regression in melanoma models	[83,84]
**Adjunct to immunotherapy**		
Transferred prepared intact mitochondria into CAR-T cells	Improved metabolic adaptability and enhanced proliferative capacity of CAR-T cells, increased cytokine production ability	[99]

Bax: BCL-2-associated X protein; Bcl-2: B-cell lymphoma 2; CAR-T: chimeric antigen receptor T cell; mtDNA: mitochondrial deoxyribonucleic acid; NIR: near-infrared; OXPHOS: oxidative phosphorylation; p-Bad: phosphorylated BCL-2-associated agonist of cell death; PINK1: PTEN-induced kinase 1; TNT: tunneling nanotube; USP30: ubiquitin-specific protease 30; α-SMA: alpha-smooth muscle actin; GES-1: gastric mucous epithelium cell line; AGS: adenocarcinoma cell line.

### Inhibition of the mitochondrial transfer pathway

Most of the aforementioned studies indicate that spontaneous mitochondrial transfer predominantly facilitates tumor development. TNTs play a crucial role in mediating the transport of mitochondria between tumor and stromal cells; therefore, inhibiting TNT formation may effectively reduce mitochondrial transfer. Possible approaches for blocking mitochondrial transfer include: (i) perturbing actin filament dynamics to suppress TNT assembly; (ii) targeting critical structural and functional components supporting TNT formation; (iii) weakening cell adhesion and shortening cell-cell contact time to minimize intercellular mitochondrial transfer; and (iv) suppressing signaling pathways that stimulate horizontal mitochondrial transfer^[42]^. Accordingly, the development of pharmacological agents aimed at disrupting the mitochondrial transfer between tumor cells and TME may represent a promising strategy for tumor inhibition. Taxanes and vinca alkaloids, frequently used as routine chemotherapy, have been shown to disrupt microtubule polymerization, thereby inhibiting mitochondrial transfer^[29]^. Given that actin is an essential structural element of TNTs, the dynamic rearrangement and polymerization of the actin cytoskeleton are fundamentally required for TNT biogenesis. In line with this mechanism, small-molecule agents targeting and disrupting actin function, including latrunculin and cytochalasin isoforms B and D, substantially suppress TNT formation and assembly^[75]^. As a key protein modulating TNT generation and a specific marker of these structures, M-sec possesses therapeutic potential. Targeting M-sec could effectively inhibit the intercellular shuttling of mitochondria^[76]^. Downregulating the expression or impairing the functional activity of M-sec significantly impedes TNTs assembly, consequently diminishing mitochondrial transportation between malignant cells^[76]^. At concentrations that exert no cytotoxic effects, L-778123, a dual inhibitor of farnesyltransferase and geranylgeranyl transferase I, markedly blocks TNTs assembly and subsequent mitochondrial exchange^[67]^. Multiple myeloma (MM) cells can resist belantamab mafodotin (belamaf), a monoclonal antibody linked to the microtubule-disrupting agent monomethyl auristatin-F (MMAF), through enhancing mitochondrial transfer from stromal cells to allow them to evade apoptosis^[77]^. Doxycycline has emerged as a novel repurposed mitochondria-targeted reagent, which can interfere with mitochondrial transfer processes and thus benefit cancer therapy^[78]^. Experimental findings reveal that administration of doxycycline enhances intercellular mitochondrial transfer among tumor cells. Such restoration of normal mitochondrial function significantly improves cancer cell responsiveness to anticancer regimens^[78]^. Optimization of mitochondrial function and cellular metabolism mediated by these drugs can normalize aberrant mitochondrial behaviors in cancer cells, ultimately boosting tumor susceptibility to clinical anticancer interventions^[78]^. Additional druggable mechanisms are GJs, which can be targeted with 18-α-glycyrrhetinic acid, and dynamin, which can be inhibited by dynasore, to suppress vesicular endocytosis^[79]^. Despite the signaling mechanisms regulating mitochondrial transfer, the identification of key molecular components, such as Miro1, Cx43, and CD38, has laid the foundation for potential therapeutic interventions^[80]^.

### Promotion of spontaneous transfer of mitochondria

In mitochondrial diseases or ischemic conditions, spontaneous mitochondrial transfer can be beneficial. For example, pretreatment of human MSCs with iron oxide nanoparticles has been shown to promote the overexpression of Cx43 and significantly enhance the efficiency of mitochondrial transfer from human MSCs to damaged alveolar epithelial cells^[81]^. Similarly, overexpression of Miro1 in MSCs potentiates the metabolic protective effects of intercellular mitochondrial transfer against neuronal oxidative damage, attributable to improved transfer efficiency^[82]^. However, in solid tumors, mitochondrial transfer may instead contribute to tumor initiation and progression. Therefore, strategies that engineer mitochondria to remain harmless to normal cells while selectively targeting tumor cells. Peng *et al*. demonstrated that inhibition of PINK1-mediated mitophagy enhances intercellular mitochondrial transfer by increasing mitochondrial motility, oxidative phosphorylation, and TNT formation. Furthermore, engineered mitochondria carrying photosensitizer IR780 can achieve deep tumor penetration via TNTs. Subsequent near-infrared irradiation induces potent photodynamic killing while disrupting mitochondrial transfer networks, resulting in up to 43% complete tumor regression in melanoma models^[83,84]^. These findings suggest that using mitochondria as delivery vehicles for therapeutic agents, combined with promoting mitochondrial transfer, represents a novel and promising strategy for cancer treatment.

### Artificial mitochondrial transplantation

Artificial mitochondrial or mitochondrial transplantation techniques have been developed and are expected to have broader applications in the future. Healthy human blood contains substantial numbers of mitochondria, suggesting a potential role in maintaining physiological homeostasis; this observation has served as the theoretical foundation for the concept of artificial mitochondrial transplantation^[85]^. These isolated mitochondria can be rapidly integrated into recipient cells, where they co-localize with endogenous mitochondria^[86]^. Currently, the initial step in mitochondrial transplantation involves isolating functional mitochondria from various cell types. Employing stem cells with high mitochondrial transfer capacity represents a viable alternative strategy for transplantation. MSC-derived mitochondria have been extensively utilized in mitochondrial therapies^[87]^. Another potential approach involves the use of induced pluripotent stem cells (iPSCs) to generate functional mitochondria suitable for transplantation. iPSCs can be derived from patients’ somatic cells and subsequently directed to differentiate into various cell types, including those affected by mitochondrial diseases^[88]^. Functional mitochondria can be produced by generating iPSCs from patients with mitochondrial disorders and subsequently transferring them to the affected tissues^[88]^. As the benefits of modifying cells to enhance effective mitochondrial transfer become increasingly evident and the therapeutic effects are progressively clarified, the use of engineered cells for mitochondrial supplementation may emerge as a promising strategy in the future^[89]^. Scientists have developed a technique known as MitoCeption, in which mitochondria are internalized by recipient cells through centrifugation-induced mechanisms and heat shock-mediated mechanisms during co-culture^[58]^. In this approach, labeled mitochondria are combined with similarly labeled recipient cells via centrifugation and subsequently co-cultured under standard conditions. During this co-culture period, recipient cells take up the isolated mitochondria, facilitating their incorporation into the cellular structure^[58]^. Another crucial step is mitochondrial infusion. Systemic administration via injection is a common method for delivering mitochondria and can be performed through routes such as the jugular or femoral veins^[90]^. Isolated mitochondria are inherently unstable; therefore, efficient delivery of exogenous mitochondria to somatic cells or tissues is essential. Recent advances in mitochondrial therapy have introduced an erythrocyte membrane vesicle-based encapsulation platform. This innovative transplantation approach substantially enhances the delivery efficiency of exogenous mitochondria into cells and tissues of mice and non-human primates^[91]^. Studies have shown that delivering mitochondria to Various types of solid tumor cells alongside low-dose chemotherapy markedly increases apoptosis in these cells and improves chemosensitivity in mouse models^[92]^. In melanoma-bearing mice, mitochondrial transplantation was found to inhibit tumor growth and lung metastasis. Transcriptomic analyses revealed a strong association between global chromosome silencing and mitochondrial activity in suppressing melanoma following mitochondrial transplantation in metastatic models^[93]^. Interestingly, mitochondria derived from female animals exhibited greater anti-tumor efficacy compared to those from males. This difference may be attributed to more robust communication between mitochondria and the nucleus mediated by female mitochondria^[93]^. Mitochondrial transplantation with intact healthy mitochondria exerts robust anti-tumor effects in hepatocellular carcinoma. This intervention suppresses tumor glycolysis, facilitates Bad dephosphorylation, modulates the B-cell lymphoma 2 (Bcl-2)/Bcl-2-associated X (Bax) balance by repressing Bcl-2 and increasing Bax expression, and eventually induces caspase-mediated apoptotic cell death in hepatocellular carcinoma^[94]^. Healthy mouse liver mitochondria were isolated and injected through tail veins into melanoma-bearing model mice, which suffered from lung metastasis. The results demonstrated that mitochondrial transplantation substantially suppressed tumor growth and extended the survival time of these mice^[95]^. The anti-tumor effects of mitochondria are linked to their ability to reprogram tumor cell metabolism by suppressing glycolysis and promoting an oxidative intracellular environment, thereby creating conditions that hinder tumor cell proliferation^[95]^. Delivery of healthy mitochondria isolated from the human normal gastric epithelial cell line GES-1 into the gastric adenocarcinoma cell line AGS effectively impairs tumor cell migration and invasion. Notably, this intervention does not alter cell viability or induce cellular apoptosis. These findings indicate that mitochondrial transplantation can effectively reduce the malignancy of gastric cancer cells^[96]^.

### Mitochondrial transplantation as an adjunct to immunotherapy

Chimeric antigen receptor (CAR) T-cell therapy is a revolutionary advancement in cellular immunotherapy, offering a personalized therapeutic strategy for cancer treatment^[97]^. Premature exhaustion of CAR-T cells serves as a critical bottleneck for therapeutic outcomes, which is closely associated with defective mitochondrial function and aberrant mitochondrial dynamic remodeling^[98]^. In addition, exogenous mitochondria have been successfully transferred into CAR-T cells in preclinical studies. This mitochondrial supplementation effectively improves cellular metabolic adaptability, enhances proliferative potential, promotes cytokine release, and significantly reinforces the anti-tumor function of CAR-T cells^[99]^.

## CONCERNS REGARDING MITOCHONDRIAL TRANSPLANTATION AS A TARGETED THERAPY

Despite the prominent therapeutic prospects of exogenous mitochondrial transplantation for tumor intervention, the clinical transformation and large-scale clinical application of this emerging therapeutic strategy still face multiple unavoidable bottlenecks and unresolved limitations. A variety of technical safety, delivery efficiency, and translational standardization barriers remain to be systematically addressed before mitochondrial transfer-targeted therapy can be safely and stably applied to clinical cancer treatment.

First, the biosafety risks associated with mitochondrial transfer intervention strategies have not been fully clarified and resolved. Small-molecule inhibitors targeting TNT formation mainly act on cytoskeletal proteins; such agents produce broad cytotoxicity against dividing normal cells and may trigger myelosuppression and organ toxicity. Moreover, these blockers indiscriminately disrupt physiological TNT-mediated mitochondrial rescue in parenchymal cells, neurons, and immune cells, disturbing tissue homeostasis, wound healing, and endogenous immune surveillance^[12]^. Notably, incomplete regulation of intercellular mitochondrial transfer may exert selective pressure on tumor subpopulations, enriching highly aggressive, drug-tolerant clones and ultimately accelerating tumor relapse. Allogeneic mitochondrial transplantation introduces additional safety and ethical concerns, as donor mitochondrial DNA may permanently integrate into recipient cells and trigger unforeseen long-term biological consequences.

Second, widespread delivery efficiency bottlenecks limit *in vivo* therapeutic performance. Isolated free mitochondria face rapid degradation and calcium-induced damage in circulating blood, while EV-based mitochondrial carriers also suffer from short systemic half-life and rapid clearance^[100]^. Dense fibrotic tumor stroma creates physical barriers that hinder deep intratumoral penetration of EVs, peptides, and small-molecule inhibitors, resulting in uneven drug distribution within tumor lesions. In addition, intratumoral heterogeneity leads to drastically variable TNT abundance across distinct tumor subclones and patient specimens. Although surface modification with “don’t-eat-me” markers (CD47, CD55, CD59, CD200) can partially reduce phagocytic clearance of EVs^[101]^, inconsistent mitochondrial loading efficiency and variable vesicle quality from different isolation methods further weaken therapeutic reproducibility^[102]^.

Thirdly, unresolved translational and standardization limitations further hinder the clinical progression of mitochondrial transfer-targeted therapy. At this stage, there is a lack of clinically validated specific imaging biomarkers and targeted detection probes that can accurately quantify TNT abundance and mitochondrial transfer activity in human tumor tissues. The absence of effective *in situ* detection techniques makes it impossible to precisely screen patients suitable for mitochondrial transplantation therapy, failing to achieve individualized and precise tumor treatment. Furthermore, the core clinical treatment specifications for mitochondrial targeted therapy have not yet been established. The optimal clinical administration dosage, safe and effective treatment intervention window, and standardized treatment course remain undefined. In addition, the synergistic application strategies of mitochondrial transfer inhibition combined with traditional clinical tumor treatment methods, including chemotherapy, radiotherapy, and tumor immunotherapy, lack systematic exploration and verification. At present, most relevant studies are limited to *in vitro* cell experiments and preliminary animal models, and there is a scarcity of standardized *in vivo* combination therapy trials to confirm the long-term stable anti-tumor efficacy and clinical safety of combined regimens, which greatly delays the clinical transformation and clinical application process of this innovative tumor therapy.

## CONCLUSIONS

The progress in mitochondrial transfer research has not only deepened our understanding of intercellular communication and interaction mechanisms but has also driven the development of novel therapeutic strategies. Despite the innovative insights brought by current technical advances, the mechanistic basis of intercellular mitochondrial transfer remains incompletely defined. Specifically, the tissue- and cell-type-specific diversity of mitochondrial transfer patterns has yet to be systematically clarified. Elucidating the exact molecular pathways through which mitochondrial transfer contributes to tumor progression, including cell proliferation, invasion, metastasis, chemotherapy resistance, and immune evasion, may reveal multiple targets for therapeutic intervention. Continuing research in this field will play a crucial role in developing more personalized and precise cancer treatments. Mitochondrial transplantation therapy for tumors remains at an experimental stage. Although promising therapeutic effects have been demonstrated in animal models, its clinical translation has not yet been achieved. Bridging the gap between laboratory research and clinical implementation presents several challenges, including safety concerns, achieving efficient delivery of mitochondria to tumor cells, and overcoming preservation-related limitations. Resolving these limitations will facilitate the clinical translation of mitochondrial transfer research and yield meaningful therapeutic gains for cancer treatment. Concurrently, novel intervention strategies targeting tumor mitochondrial transfer are under continuous investigation. Early successes of this approach bear considerable potential, indicating that targeting metabolic interactions among tumor cells could represent a novel approach to suppress tumor growth and metastasis.

Accumulating evidence suggests that artificial transfer of mitochondria into tumor cells can exert either pro-tumorigenic or anti-tumor effects, depending on the context. Determining how to make reliably direct artificially transplanted mitochondria toward tumor-inhibitory outcomes under specific conditions remains a critical challenge that warrants further investigation.

## DECLARATIONS

### AI and AI-assisted tools statement

During the preparation of this manuscript, the AI tool DeepSeek (version V3, released 2024-12-26) was used solely for minor language revision. The tool did not influence the study design, data collection, analysis, interpretation, or the scientific content of the work. All authors take full responsibility for the accuracy, integrity, and final content of the manuscript.

### Copyright

© The Author(s) 2026.
